# Absent cervical spine pedicle and associated congenital spinal abnormalities - a diagnostic trap in a setting of acute trauma: case report

**DOI:** 10.1186/1471-2342-10-25

**Published:** 2010-11-09

**Authors:** Roman Guggenberger, Gustav Andreisek, Hans Scheffel, Simon Wildermuth, Sebastian Leschka, Paul Stolzmann

**Affiliations:** 1 Diagnostic and Interventional Radiology, University Hospital Zurich, Zurich, Switzerland; 2 Institute of Radiology, General Hospital Saint Gall, Saint Gall, Switzerland

## Abstract

**Background:**

Congenital spinal abnormalities can easily be misdiagnosed on plain radiographs. Additional imaging is warranted in doubtful cases, especially in a setting of acute trauma.

**Case Presentation:**

This patient presented at the emergency unit of our university hospital after a motor vehicle accident and was sent to our radiology department for imaging of the cervical spine. Initial clinical examination and plain radiographs of the cervical spine were performed but not conclusive. Additional CT of the neck helped establish the right diagnosis.

**Conclusion:**

CT as a three-dimensional imaging modality with the possibility of multiplanar reconstructions allows for the exact diagnosis and exclusion of acute traumatic lesions of the cervical spine, especially in cases of doubtful plain radiographs and when congenital spinal abnormalities like absent cervical spine pedicle with associated spina bifida may insinuate severe trauma.

## Background

Absent cervical spine pedicle (ACSP) is a very rare congenital abnormality of the spine. It has been described first in 1946 by Hadley [1-4] and is characterized by the absence of a pedicle of the affected vertebral body. ACSP may be associated with other congenital osseous abnormalities of the cervical spine [2,5,6]. Since most patients are asymptomatic for many years, the majority of them remain undiagnosed until neck injury, pain or paresthesias in the neck or arm warrant an imaging evaluation. In a setting of acute trauma however, ACSP can be a source of radiologic misdiagnosis i.e. on plain radiographs.

## Case Presentation

Following a motor vehicle accident, a 38 year-old male patient presented at the emergency room of our hospital. He was alert and in stable condition with a GCS-score of 15. On clinical examination the patient showed mild neck tenderness to palpation as well as a slightly restricted range of motion. No sensomotor deficits of the neck or arms were present. The patient commented that he had been suffering from similar neck pain for many years prior to the accident.

**Figure 1:**
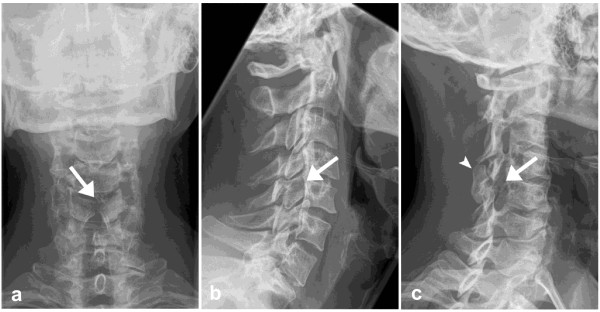
**a-c: Plain Radiographs of the cervical spine**. Antero-posterior-, lateral- and oblique-view radiographs of the cervical spine: A vertical gap of the C5 arch is seen on the antero-posterior-view radiograph (a). On lateral-view (b) slightly incongruent articular pillars of C5 are noted. On an oblique-view radiograph of the right side (c) an enlarged and elongated right C4-C5 neuroforamen with absence of the right C5 pedicle is seen. The opposite lamina projects through this space (arrow). There is also dorsal displacement of the articular pillar (arrowhead).

According to Canadian C-Spine rules and due to the mechanism of the accident radiography of the cervical spine was indicated. Antero-posterior and lateral radiographs as well as 45° oblique views of the cervical spine were taken as a first imaging study (Figure 1). Radiographs showed slightly incongruent articular pillars of C5 and an enlarged, elongated right C4-C5 neuroforamen with absence of the right C5 pedicle. The opposite lamina projected through this space and the articular pillar was dorsally displaced. In addition, a vertical gap in the midline of the C5 arch was seen on the antero-posterior radiograph. No anterolisthesis of a vertebral body was detected. Independently from each other, both the emergency medicine physician as well as the radiology resident on-call suspected a fracture of the right pedicle and median arch of C5. The patient was therefore scheduled for an emergency operation.

**Figure 2:**
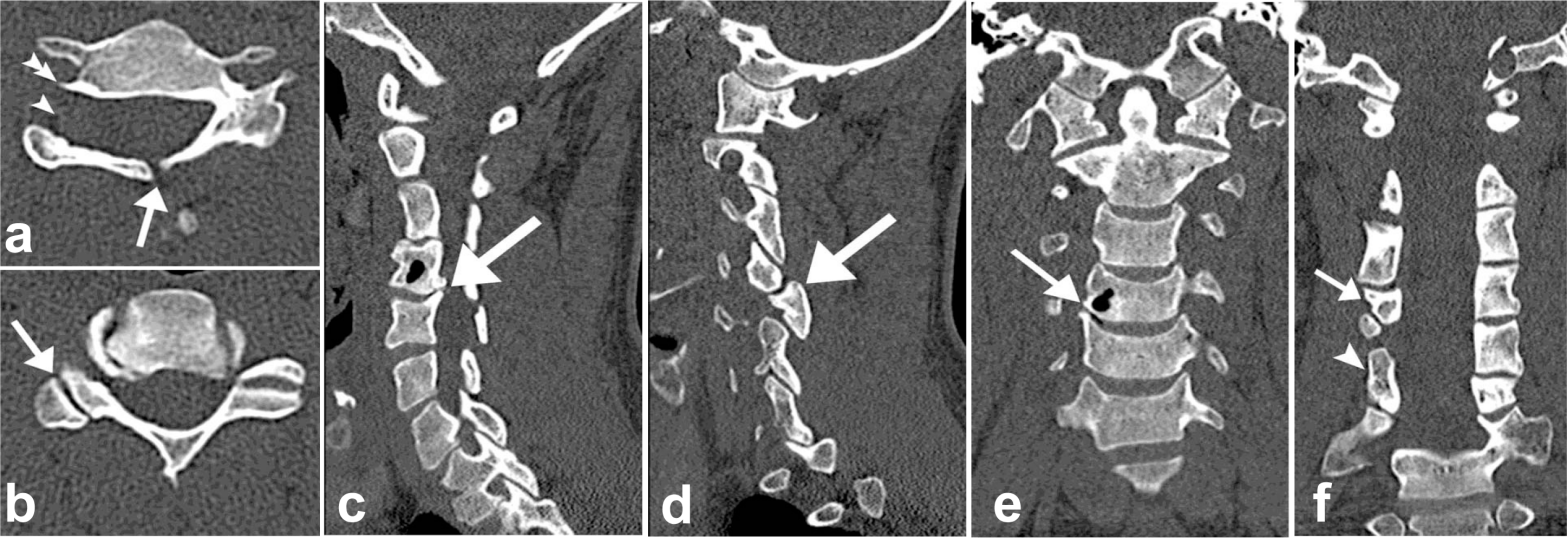
**a-f: Computed Tomography of the cervical spine**. Axial computed tomography (CT) images (a, b) and reconstructed images in the sagittal (c, d) and coronal (e, f) planes. Axial images at the level C5 show absent right cervical pedicle (arrowhead in a), spina bifida occulta (arrow in a) and dysplasia of the ipsilateral transverse process (double arrowhead in a) as well as reversed facet-joint (arrow in b). Images in the sagittal (c, d) and coronal (e, f) planes show degenerative changes at the level C4-5 (arrow in c) with formation of a bone cyst in C4 (arrow in e). The dorsal displacement of the articular pillar and reversal of the ipsilateral facet articulation (arrow in d) as well as hypoplasia of the pillar of the supra-adjacent (arrow in f) and hyperplasia of pillar of the infra-adjacent vertebra (arrowhead in f) are also well appreciated on the coronals.

**Figure 3:**
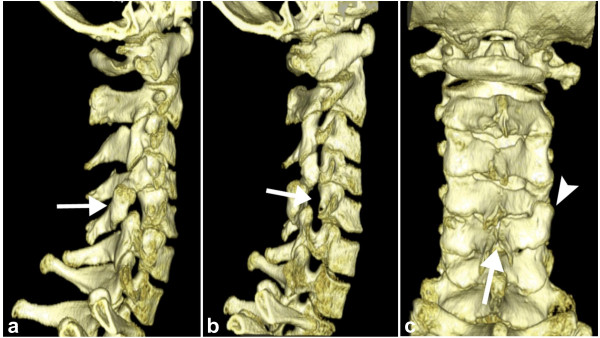
**a-c: Volume Rendering Technique - Images of cervical spine**. Lateral view VRT images illustrate the dorsally displaced right articular pillar of C5 (arrow in a). The abnormal enlarged right intervertebral foramen of C4-5 (arrow in b) which is a consequence of the absent cervical pedicle is displayed on an oblique view VRT image. Spina bifida occulta at the same level (arrow in c) as well as a reversed facet-joint on the right (arrowhead in c) are well depicted on a dorsal view VRT images (c).

According to established guidelines at our hospital, computed tomography (CT) with multiplanar reconstructions in the sagittal and coronal plane was performed preoperatively. It revealed absence of the right C5 pedicle with dysplasia of the ipsilateral transverse process and spina bifida occulta at the same level, dorsal displacement of the articular pillar, reversal of the ipsilateral facet articulation with the supra-adjacent vertebra, as well as hypoplasia of the pillar of the supra-adjacent and hyperplasia of the pillar of the infra-adjacent vertebra (Figure 2, 3). There were also some degenerative changes at the level C4-C5 with formation of a subchondral bone cyst in the body of C4 (Figure 2). No anterolisthesis of a vertebral body, fracture or hematoma were detected. Based on all CT imaging findings, diagnosis of a congential ACSP with associated osseous spinal abnormalities was established. The patient was discharged from the emergency room without surgical intervention.

He was informed and gave informed consent that data concerning his case would be submitted for publication.

## Discussion

In this case, congenital ACSP of C5 with associated osseous spinal abnormalities in a male patient who has had a motor vehicle accident was misdiagnosed as a cervical fracture on plain radiographs. The correct diagnosis could only be established by the means of CT. In their study from 2005 Holmes et al. found a pooled sensitivity for cervical spine injuries on plain radiography of 52% (95% CI 47, 56%) and for CT of 98% (95% CI 96, 99%) [7]. Despite the absence of randomized controlled trials, evidence exists that CT significantly outperforms plain radiography as a screening test for patients at very high risk of cervical spine injury. There is not sufficient evidence though to suggest that cervical spine CT should replace plain radiography as the initial screening test for less injured patients who are at low to moderate risk for cervical spine injury but still require a screening radiographic examination, as in our case. Although arguably primary CT-scan of the spine would have been the first choice in this setting, antero-posterior and lateral radiographs as well as 45° oblique views of the cervical spine were taken as a first imaging study (Figure 1).

ACSP is a very rare congenital abnormality of the spine. Any level of the cervical spine can be affected, though ACSP has been seen most frequently at the level C6 followed by the level C5 and C7 [1-5]. Although the exact pathogenesis of ACSP is unclear, it probably relates to in-utero defects in the formation of the chondrification and/or ossification centers of the spine.

In our case, ACSP was associated with several congenital osseous abnormalities. In previous reports, associations of ACSP with hypoplasia of the pedicles, hypoplasia of the vertebral body, sagittal vertebral body clefts, vertebral body and arch fusions or spina bifida occulta at the absent pedicle level have been described in up to 51% of all cases [4]. Interestingly, hyperplasia of the contralateral pedicle at the involved level has not been encountered in the literature, as is commonly seen in absent or hypoplastic lumbar pedicle [8]. In our patient, ACSP of C5 in association with spina bifida occulta at the same level resulted in a right vertebral hemi-arch with no osseous attachment to the adjacent vertebral bones. This has, to the best of our knowledge, not been reported in the radiologic literature so far. Ligaments of the spine and neck muscles might have maintained the anomalous fragment in its position, but deviation upon movement may inevitably have provoked the chronic symptoms of the patients [2,4,9]. Degenerative osseous changes of the involved or adjacent vertebral segment are frequently seen and a result of abnormal forces on the bones and articulations [4].

Antero-posterior and lateral-view radiographs of the cervical spine as a first imaging work-up give valuable information on the gross anatomy and the alignement of the vertebral structures, although subtle pathologies, e.g. fracture, of the vertebral pedicles cannot always be sufficiently ruled out [10]. Therefore many centers add oblique-view projections to their standard-views of the spine to better depict the intervertebral neuroforamina and their surrounding structures, especially in a setting of acute trauma [11,12]. Even with that extra-information it is sometimes difficult to distinguish congenital abnormalities of the spine, e.g. ACSP or spina bifida occulta, from acute traumatic injuries such as fractured pedicles or vertebral arch fractures [13-15]. CT as a three-dimensional imaging modality with the possibility of multiplanar reconstructions allows for the exact diagnosis of acute traumatic lesions of the cervical spine. It also permits to reliably identify congenital osseous abnormalities such as ACSP as well as to narrow the differential diagnosis of pathologies that might cause similar appearances on radiographs (e.g. neurofibroma which can also cause an enlarged intervertebral neuroforamen) [3]. Thereby any unwarranted surgery or inadequate conservative therapy can be avoided.

## Conclusion

The knowledge of rare congenital osseous abnormalities of the spine such as ACSP as well as of their typical radiographic appearance is essential for correct diagnosis. Especially in a setting of acute trauma when radiography is indicated, CT should be considered as primary choice to establish diagnosis and prevent misinterpretation of congenital abnormalities on plain radiographs.

## Competing interests

The authors declare that they have no competing interests.

## Authors' contributions

RG performed the literature search and compiled data presented in this report. GA and HS provided the expertise for selective imaging and contributed to the diagnosis. SW and SL conceived of the study, and participated in its design and coordination and helped to draft the manuscript. PS provided intellectual input and critically revised the manuscript. All authors read and approved the final manuscript.

## Consent

Written informed consent was obtained from the patient for publication of this case report and any accompanying images.

## Pre-publication history

The pre-publication history for this paper can be accessed here:

http://www.biomedcentral.com/1471-2342/10/25/prepub
